# A Comprehensive Systematic Scoping Review of Preventive Medicine and Public Health Systems in Saudi Arabia in the Context of Vision 2030: Review of Public Health System and Preventive Medicines in Saudi Arabia

**DOI:** 10.31661/gmj.v14i.3692

**Published:** 2025-01-09

**Authors:** Ghanim Hamid Al-Khattabi, Akshay Kiran

**Affiliations:** ^1^ Preventive Medicine and Public Health Department, Makkah Healthcare Cluster, Ministry of Health, Saudi Arabia; ^2^ Epidemiology Department, Makkah Healthcare Cluster, Ministry of Health, Saudi Arabia; ^3^ Population Health Management Department, Makkah Healthcare Cluster, Ministry of Health, Saudi Arabia; ^4^ The Transformational Healthcare Projects Department, Makkah Healthcare Cluster, Ministry of Health, Saudi Arabia; ^5^ The Enterprise Risks Management Department, Makkah Healthcare Cluster, Ministry of Health, Saudi Arabia; ^6^ The Saudi Board of Preventive Medicine Department, Makkah Healthcare Cluster, Ministry of Health, Saudi Arabia; ^7^ The Chief Healthcare Excellence Executive Offices, Makkah Healthcare Cluster, Ministry of Health, Saudi Arabia; ^8^ Department of Scientific Clinical Research, National Institute of Medical Science, Acharya Nagarjuna University, Andhra Pradesh, West Godavari District, India; ^9^ Department of General and Alternative Medicine, National Institute of Medical Science, Acharya Nagarjuna University, Andhra Pradesh, West Godavari District, India; ^10^ Department of Hospital and Health Care Administration, National Institute of Medical Science, Acharya Nagarjuna University, Andhra Pradesh, West Godavari District, India; ^11^ Department of Scientific Medical Journal Publication, National Institute of Medical Science, Acharya Nagarjuna University, Andhra Pradesh, West Godavari District, India

**Keywords:** Public Health, Health Systems, Preventive Medicine, Health Outcomes, Saudi Arabia, Vision 2030, Scoping Review, Systematic Review, Review Literature as a Topic

## Abstract

**Background:**

The Kingdom of Saudi Arabia’s Vision 2030 seeks to improve public health and healthcare but struggles to include preventative medicine. Sustainable development, innovation, and preventative care are prioritized to build a society with enough healthcare assistance. The purpose is to improve people’s health. This study examined how recent improvements in the Saudi Arabian public health system have affected preventative medicine, health justice, and public health.

**Materials and Methods:**

This scoping review examined Saudi Arabia’s public health system and preventive medicine, following Arksey and O’Malley. Vision 2030 reforms are prioritized, and systematic searches, research selection, and theme analysis improve health equity and population health. All is done according to PRISMA-ScR. Inclusion criteria included studies addressing public health system reforms in Saudi Arabia, focusing on preventive medicine initiatives, assessment of health outcomes, original research, and published between January 2017 and January 2023.

**Results:**

The review focused on the healthcare delivery model, preventative services, public health policies, and interventions. Saudi Arabia’s public health strategies focus on improving healthcare delivery through telehealth, combining preventive services like health education and access to preventive care, controlling infectious diseases and involving the community, educating the public, and getting the government involved to improve results.

**Conclusion:**

Overall, Saudi Arabia has improved health intervention outcomes, public health policies, healthcare delivery techniques, and preventative programs. Policymakers, healthcare workers, and researchers can use these findings to improve public health. It ends by identifying research topics and areas for further study to support the Kingdom’s Vision 2030 aims.

## Introduction

Vision 2030 has accelerated development in several areas, including the health system, to diversify Saudi Arabia. Improve resident and tourist well-being to achieve this goal. The country’s health care system can prioritize primary care, infectious and non-communicable diseases, and more. Prevention has enormous potential to reduce health risks and improve overall health. This scoping assessment aims to provide a complete picture of Saudi Arabia’s public health and preventive medicine, including its current state, past achievements, current issues, and future potential.

## Search Strategy

The approach used to search for this scoping review was informed by the desire to find any literature available on the public health system and preventive medicine in Saudi Arabia. Due to this, a multiple database search was conducted using the Arabic Search Index, Al Manhal, Dar Al-Mandumah, Saudi Digital Library, Google Scholar, and Qatar National Library Digital Resources. Researchers examined Saudi Arabia’s public health infrastructure, non-communicable diseases, preventive medicine, and the health initiatives outlined in Vision 2030. The book was slated for publication from January 2017 to December 2023.

The search terms were Saudi Arabian healthcare system, primary care, public health services, preventative medicine, non-communicable disease reduction, and Saudi Vision 2030’s health system reforms.

Boolean operators (AND, OR) improved the primary search method and found studies that covered all four main issues in detail (Hasan et al., 2021). We checked the reference lists of all the papers we read to ensure a comprehensive search. We searched for relevant studies that were not in our databases.

These criteria were defined beforehand to minimize bias in the search for studies. The study included only articles published in English-referred journals. For this purpose, only papers that discussed the public health system of the Kingdom of Saudi Arabia (KSA), primary care services, preventive medicine programs, or Vision 2030 for KSA were considered (Khan et al., 2021). Excluded studies were those reporting clinical medicine without a public health component, commentaries, and non-peer-reviewed publications.

These were complemented by manual searches of the key government and non-governmental organization (NGO) reports, including those of the Saudi Ministry of Health, the World Health Organization (WHO), and all other organizations that may have some influence on the Saudi health systems (Aljunayd et al., 2020). This added an extra layer of filtering and safeguarded the search to cover all sides of the efforts in public health in the Kingdom.

## Study Selection

A meticulous screening process ensured the inclusion of just the most pertinent and high-calibre studies. A total of 1,032 works were identified across all databases following the search. The selection process comprised three components;

### Title and Abstract Screening

Initially, publication titles and abstracts were evaluated to exclude those not meeting the criteria (Aljadeed et al., 2021). Two researchers evaluated the items separately for eligibility. The systematic search omitted papers that did not address public health or prevention (Khan et al., 2021). This narrowed the study pool to 284 manuscripts.

### Full-text Review

In the second stage, the full texts of the chosen items were retrieved to do more research. Aljunayd et al. (2020) reported that there are stringent inclusion and exclusion criteria throughout this phase. We did not include any studies that did not provide information regarding Saudi Arabia, primary healthcare, disease prevention, or improvements to the healthcare system. We did not include any stories that did not refer to Saudi Arabia (Khan et al., 2021). During this round, 102 investigations passed on to the data analysis stage.

### Final Selection

The authors began by searching the existing literature for primary sources that (Aljadeed et al., 2021) provided information on the public health system and illness prevention in Saudi Arabia. After eliminating papers with sufficient datasets or lacking public health performance criteria, forty research studies were chosen for the scoping review examination. A third party intervened if the initial two judges could not reach a consensus (Al-Worafi, 2020). Once the studies had been chosen, they were categorized according to the following topics: public health sector organization, primary care, preventive medicine, and changes in healthcare due to Vision 2030.

## Data Extraction

**Figure-1:**
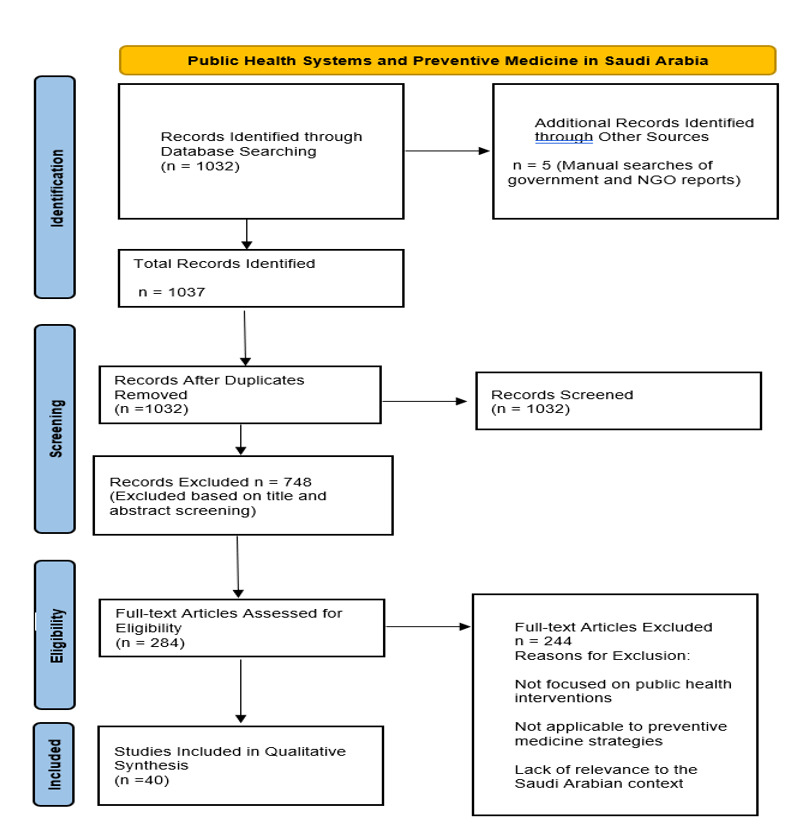
Figure 1. Preferred reporting items for systematic reviews and Meta-analyses (PRISMA) flow diagram showing the results of a search conducted for public health systems and preventive medicine literature in Saudi Arabia (January 2017 – December 2023

Research data collection: utilized a tried-and-true, pilot-tested form to ensure comprehensive data collection (Hasan et al., 2021).

Here are some crucial points:

1. Information about the study, including the authors, publication date, methodology, location, and sample size.

2. Alterations to health-related habits, disease, or mortality are some health consequences that could result from these shifts.

3. Primary care includes curative and preventative treatments and sharing relevant information.

4. Preventive medicine includes vaccinations, health education, disease management programs, and health promotion.

5. The impact of Vision 2030 on healthcare policy changes and their impact on services.

Issues and potential remedies: Opportunities for expansion, challenges in resource allocation, and difficulties in providing services are all present.

This enabled the compilation of all data about the health development projects in Saudi Arabia (Aljunayd et al., 2020). Another researcher double-checked the results to ensure their accuracy.

## Results

**Figure-2:**
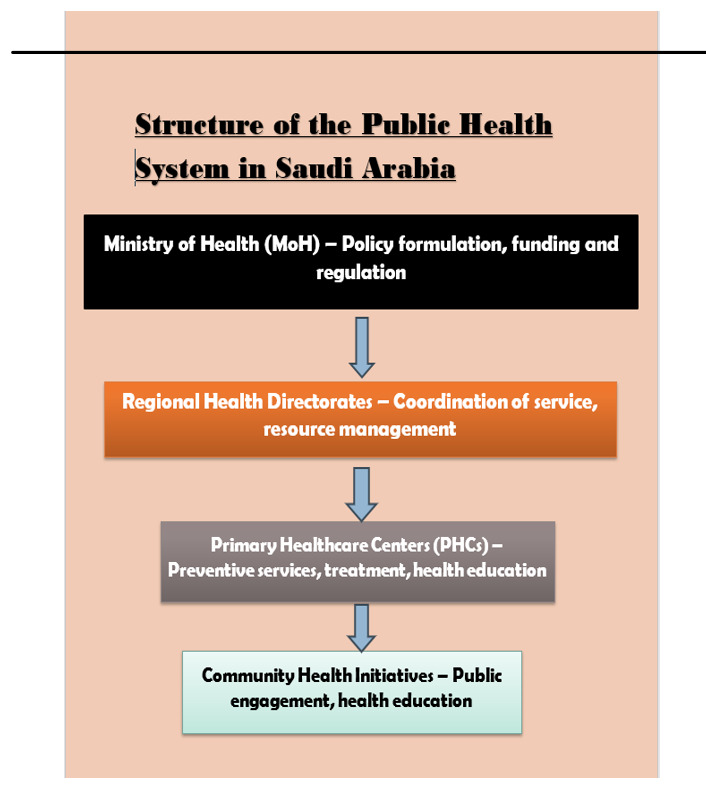
Figure 2. Structure of the public health system in Saudi Arabia

### Structure of the Public Health Systems

The review exposed that there has been a process of structural changes in Saudi Arabia’s public health system, especially regarding Vision 2030(Aljadeed et al., 2021). The Ministry of Health (MoH) continues to lead the formulation and implementation of health policies, financing healthcare services, and overseeing healthcare institutions (Asmri et al., 2020). The system is divided into three levels of care: the first division of services includes primary, secondary, and tertiary services, with primary health care as the initial interface for providing services.

Several works discussed the issues of resource mobilization for health and capacity strengthening to enhance access to health services and health care outcomes at the first level of care. Al Khashan et al. (2021) posited that the Saudi government had increased efforts to enhance physical support and access to critical drugs in primary care centers. However, the distribution of resources, especially in rural health facilities, has remained challenging due to significant differences between rural and urban settings (Al Saffer et al., 2021).

### Health Promotion Efforts

Preventive healthcare promotion has been adopted in Saudi Arabia mainly due to the growing incidence of NCDs like diabetes, hypertension, and cardiovascular diseases (Chowdhury et al., 2021). The review identified that health education, early detection, and lifestyle changes have been vital in reducing disease burden (Adly et al., 2020). Preventive care, especially for chronic conditions, has been one of the most successful strategies for identifying and encouraging at-risk populations to get checked.

The preventive programs have also increased productivity under Vision 2030(Adly et al., 2020). Health promotion activities, including vaccination and health awareness, were identified in several papers as strategies adopted to prevent both communicable and non-communicable diseases (Alasiri & Mohammed, 2022). Another shift was the sparing of telehealth services during the COVID-19 pandemic, which allowed people to receive preventive care in remote environments (Alghamdi et al., 2020). identified that health education, early detection, and lifestyle changes have been vital in reducing disease burden (Adly et al., 2020). Preventive care, especially for chronic conditions, has been one of the most successful strategies for identifying and encouraging at-risk populations to get checked.

### COVID-19 Impact

The review under consideration also revealed the effects of COVID-19 on the Kingdom of Saudi Arabia’s public health system. During the pandemic, organizational resources and their availability were instrumental in addressing emergencies (Al-Worafi, 2020). Almutairi et al. (2020) noted that public adherence to preventive measures was vital to preventing the virus’s spread. State initiatives were instrumental in raising awareness regarding vaccination for COVID-19 and measures like wearing masks and social distancing.

HoweIt was also revealed that there is a long way to go in developing health facilities, especially in rural areas, regarding the availability of COVID-19 treatment and preventive measures (Rahman & Al-Borie, 2021). These challenges highlighted the problem of the underdevelopment of relevant capacities necessary to adequately prepare for and respond to following system shocks.

## Discussion

Vision 2030 prompted the scoping evaluation of forty research articles on Saudi Arabia’s public health system and preventative medicine. These studies covered (Adly et al., 2020) primary care, healthcare reforms, methodology, system transformation, and problems and opportunities.

Public Health System Transformations: Many studies have examined structural and organizational changes. Vision 2030 aims to improve governance by creating an integrated, efficient system prioritizing public health and primary care. The study stressed the need for better healthcare service coordination, increased health insurance, and equitable resource distribution to impoverished and rural regions (Al-Qahtani et al., 2020).

Primary care and preventive medicine measures like health education, disease prevention, and immunization drives have been extensively studied. Early disease detection, immunization, and lifestyle change were part of Vision 2030’s goal to reduce non-communicable diseases (Alyabsi et al., 2020). Research has also shown that integrating preventative medicine into primary care improves holistic health management.

Numerous publications evaluated the effects of Vision 2030 healthcare reforms (Al-Qahtani et al., 2020), including privatization and innovative care models to improve accessibility and efficiency. Healthcare delivery is changing due to developments in digital health technology, including telemedicine, electronic health records, and public-private partnerships.

## Challenges and Opportunities

**Figure-3:**
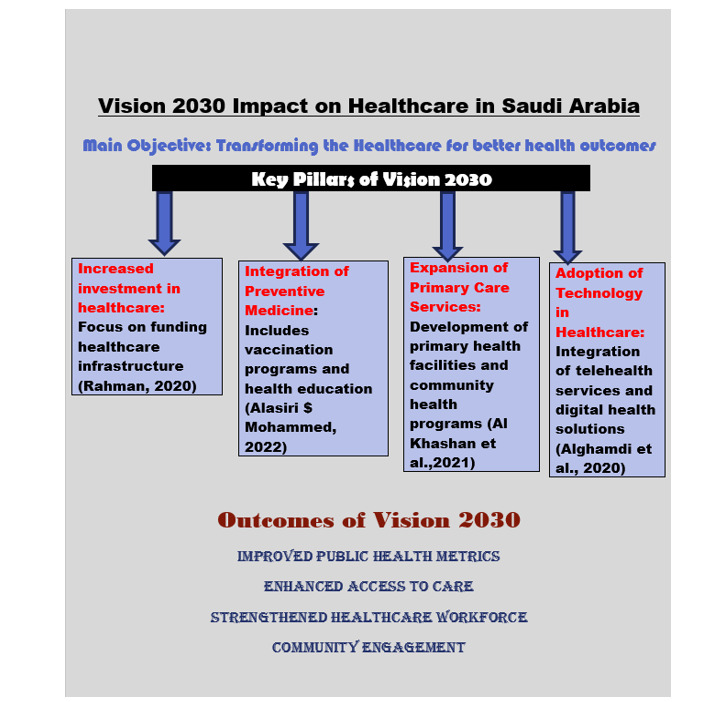
Figure 3. Vision 2030 impact on healthcare in Saudi Arabia

### Key Challenges

The scoping study found that the Saudi public health system and preventive medicine face major issues that make achieving Vision 2030 targets difficult. These issues include cultural differences, a shortage of skilled staff, incompatible data systems, and rising NCD rates (Alyabsi et al., 2020).

Initially, unbalanced resource allocation persists. Due to the concentration of healthcare centres, medical supplies, and qualified staff in cities, rural and outside residents sometimes have trouble accessing them (Alshammari et al., 2020). Due to this discrepancy, Vision 2030 goals like fair health outcomes and universal health care cannot be achieved.

The second issue is a shortage of doctors and other medical professionals, particularly in public health and preventive medicine (Aljadeed et al., 2021). Lifestyle-related disorders are increasing, making the already busy workforce more challenging to manage. To solve this issue, focus on hiring, training, and retaining top talent.

Poor public health data system integration is another major issue. Without reliable, uniform data, healthcare and preventative initiatives cannot be planned, monitored, or evaluated (Rahman & Al-Borie, 2021).

The lack of integrated data tools makes tracking health trends and their effects more challenging. Thus, fact-based decision-making slows.

Culture and behaviour may make public health goals difficult. Health promotion programs fail because people do not know how to stay healthy, do not want to change their lifestyle, and are dubious about immunizations (Adly et al., 2020). Public health programs can assist communities in solving these issues by understanding cultures, engaging with communities, and promoting permanent behavioural changes.

Finally, rising rates of non-communicable diseases (NCDs) like diabetes, obesity, and heart disease are a significant issue. These diseases, primarily caused by poor diet, inactivity, and smoking, strain healthcare systems. Preventative measures (Aljunayd et al., 2020), including health education, early detection, and community involvement, can reduce this burden.

Vision 2030 has improved Saudi Arabia’s public health, but there is still a long way to go. More healthcare personnel, better data integration, preventative treatment, and fairer resource allocation are needed to sustain the healthcare system and keep it financially solvent (Aljadeed et al., 2021).

### Opportunities for Improvement

Saudi Arabia’s public health system and preventative medicine have issues, but there is also much potential to achieve Vision 2030. To overcome these issues, the healthcare system must invest wisely, reform policy, and innovate.

First, better resource distribution can make healthcare more accessible, especially in rural and underprivileged areas. By fairly distributing medical supplies, equipment, and qualified experts, the government may ensure all communities have access to high-quality healthcare. Mobile health centres and telemedicine must be funded to provide medical treatment to everyone. Vision 2030 seeks better services and health insurance for all. This would simplify our goal (Aljadeed et al., 2021).

Second, healthcare personnel improvement is crucial. Undergraduate and graduate public health and preventative care programs are crucial. Increasing medical education, offering professional development, and encouraging healthcare professionals to work in neglected areas (Alshammari et al., 2020) will help solve the shortage of healthcare personnel. While local professionals are trained, experienced healthcare workers from other nations may be needed to satisfy acute demands.

Healthcare data integration also needs improvement.

A nationwide electronic health record system integrating public and private patient data could improve treatment coordination and public health trends (Al-Qahtani et al., 2020). This will allow health professionals to evaluate preventative programs, personalize treatments, and make sensible public health project decisions.

If we target health education, we may be able to overcome habits and culture. Healthy living choices, frequent check-ups and immunizations, and exercise are recommended to improve behaviour (Alghamdi et al., 2020). Community-based health programs and media campaigns that are culturally sensitive can influence people’s views about health interventions and increase their acceptance.

Finally, Saudi Arabia can reduce NCD rates by emphasizing early diagnosis and prevention. Public health programs encouraging smoking cessation, exercise, and healthy eating help reduce non-communicable diseases (NCDs) (Al-Qahtani et al., 2020). Adding preventative treatments to primary healthcare will enhance long-term health outcomes, cut costs, and ensure lifelong care.

## Points for Practitioners

Doctors and nurses should remember these as Saudi Arabia works toward its Vision 2030 public health and preventative medicine goals:

*Healthcare practitioners should promote preventive screenings, vaccines, and check-ups. Early detection of cancer, high blood pressure, and diabetes may improve patient outcomes and save healthcare costs. Adding preventative treatments to general care ensures long-term care.

*Telemedicine, EHRs, and mobile health apps can improve healthcare. Doctors should use these tools in locations without enough doctors to monitor patients, speed up communication, and give remote consultations. This would aid Vision 2030’s goal of digitally changing healthcare.

*Health educators should utilize culturally appropriate ways to help people adopt healthy habits. Health education initiatives should meet people’s cultural ideas and conventions to increase acceptance. Healthcare providers can dramatically reduce non-communicable diseases (NCDs) by promoting healthier food, more significant activity, and smoking cessation programs.

*Healthcare workers should collaborate more with education, social services, and urban planning to address socioeconomic variables that affect health. This comprehensive approach can improve public health and reduce the healthcare system’s workload by identifying the core causes of health issues.

*Healthcare personnel must constantly learn to keep up with industry developments. Continuing education and research on public health policy, preventative measures, and new health challenges can help providers adapt to the ever-changing healthcare system.

*To ensure rural and impoverished patients receive care, healthcare professionals should advocate for better resource distribution. One example is pushing for relocating people, tools, and medical facilities from underserved areas.

*Doctors should routinely use health data to analyse outcomes, discover trends, and evaluate therapies to enhance patient outcomes. Practitioners can improve public health policies and initiatives by participating in national data gathering and maintaining accurate patient records.

As Saudi Arabia progresses toward Vision 2030, privatizing some services, healthcare providers should engage with private enterprises to (Al-Qahtani et al., 2020) improve care while keeping quality and pricing low. School, illness prevention, and service improvement partnerships could achieve this.

Health practitioners should educate people about healthy eating (Puteh et al., 2020), exercise, and lifestyle modifications to reduce non-communicable diseases (NCDs). Working with local groups can assist health education initiatives in reaching more people and encouraging self-care to prevent chronic diseases.

## Study Limitations

The study’s main shortcoming is the lack of public health statistics. Saudi Arabia’s healthcare system analysis is unreliable without a comprehensive data repository. Due to data quality differences between rural and urban areas, population-wide conclusions are difficult (Rahman & Al-Borie, 2021). Data-driven healthcare must bridge this gap.

The size of the study was another issue. The review solely examined English-language literature, so it may have missed Arabic studies on regional cultural and public health issues (Hasan et al., 2021). Many public health measures use government and NGO reports.

These reports may have been excluded because other experts did not review them (Aljunayd et al., 2020).

Also troublesome was population representation. The big city healthcare policy and trends study excluded rural individuals. Healthcare access and outcomes, especially preventative treatments, depend on resource distribution (Al Saffer et al., 2021).

Another flaw is that the study only used research from 2017 to 2023. Despite starting at the same time as Vision 2030, this period may not include all key historical events or long-term healthcare developments (Aljadeed et al., 2021). Adly et al. (2020) claim the study is insufficient since it lacks cultural and behavioural factors, including vaccine resistance and risky lifestyle choices.

## Future Research Concepts

Integrated health data systems should be studied to tackle these concerns. According to Rahman and Al-Borie (2021), a national database of urban and rural public health data will assist researchers in making better decisions and performing more extensive studies. Geographical statistics can also assist policymakers in addressing healthcare inequality, said Al Saffer et al. (2021).

Additional Arabic and local language books are needed. According to Hasan et al. (2021), this would provide a more complete picture and ensure that research covers culturally unique concerns and solutions.

Future research should examine remote health care.

To ensure equal healthcare access for everybody, we must study marginalized groups’ resource and access challenges (Alshammari et al., 2020).

Vision 2030’s long-term effects require longitudinal research. This study may reveal healthcare system improvements and preventative medicine program efficacy (Aljadeed et al., 2021).

Cultural and behavioural change throughout time should guide the study. Health education programs and interventions must grasp how social behaviors affect prevention (Adly et al., 2020).

The Vision 2030 project outputs must be carefully analysed. We must evaluate their efficacy in improving digital health integration and privatization (Al-Qahtani et al., 2020).

The effects of digital health instruments are another exciting subject for study. Alghamdi et al.’s 2020 study on how digital tools affect preventative healthcare delivery, especially in marginalized areas, may inspire new programs.

Finally, future research should prioritize public participation. Alasiri and Mohammed (2022) suggest researching culturally relevant programs and community-based health education to promote prevention.

## Conclusion

Saudi Arabia’s public health system and preventive medicine programs face cultural issues, a lack of qualified staff, data fragmentation, resource inequality, and rising non-communicable disease rates. However, Vision 2030 strategies can grow. Equitable resource distribution, hiring more healthcare workers, and adopting new technology like telemedicine and digital health can improve Saudi Arabia’s healthcare system.

Health education and public health activities must be culturally relevant to promote wellness and prevention. Collaboration between businesses to address socioeconomic variables affecting health is another way to improve public health. Healthcare workers should promote equitable resource allocation, evidence-based decision-making, and lifelong learning to improve patient care.

These opportunities may improve Saudi Arabia’s healthcare system’s sustainability, equity, and prevention. Physicians, policymakers, and communities working together to implement Vision 2030 healthcare changes would improve national health.

## Conflict of Interest

None.
